# The SYSCILIA gold standard (SCGSv1) of known ciliary components and its applications within a systems biology consortium

**DOI:** 10.1186/2046-2530-2-7

**Published:** 2013-05-31

**Authors:** Teunis JP van Dam, Gabrielle Wheway, Gisela G Slaats, Martijn A Huynen, Rachel H Giles

**Affiliations:** 1 Centre for Molecular and Biomolecular Informatics, Radboud University Medical Centre, Nijmegen 6500 HB, The Netherlands; 2 Section of Ophthalmology and Neuroscience, Leeds Institute of Molecular Medicine, Wellcome Trust Brenner Building, St James’s University Hospital, Leeds LS9 7TF, UK; 3 Department of Nephrology and Hypertension, University Medical Center Utrecht, Utrecht 3508 GA, The Netherlands

## Abstract

The multinational SYSCILIA consortium aims to gain a mechanistic understanding of the cilium. We utilize multiple parallel high-throughput (HTP) initiatives to develop predictive models of relationships between complex genotypes and variable phenotypes of ciliopathies. The models generated are only as good as the wet laboratory data fed into them. It is therefore essential to orchestrate a well-annotated and high-confidence dataset to be able to assess the quality of any HTP dataset. Here, we present the inaugural SYSCILIA gold standard of known ciliary components as a public resource.

## Review

High-throughput (HTP) experiments and their computational analyses are becoming increasingly important as basic fundamental research tools. However, concerns have been raised with respect to the quality of the earliest comparative analyses of genomics data [1]. For example, the quality of HTP experiments and their bioinformatic analyses is typically undocumented and indeed often unknown. Quality, sensitivity and accuracy are important parameters to consider when deciding how to carry out HTP methods, determine cut-off thresholds and objectively evaluate the results. Within the SYSCILIA consortium, we aim to systematically evaluate the quality of our HTP experiments, such as genome-wide siRNA screening, as well as develop powerful bioinformatic tools and analytical tools to exploit the large datasets produced by HTP procedures across multiple centers. Here, we present one such tool we have generated, the SYSCILIA gold standard (SCGS) of known ciliary genes.

The SCGS is a standardized list of verified ciliary genes, which can be used as a reference dataset of cilia genes for quality metric analyses of experiments, and analyses investigating the cilium and its components. This list is not meant to be comprehensive but rather to be highly reliable; we err on the side of caution to ensure that the genes in this publically available list all encode well-characterized ciliary components. Such a gold standard is a very powerful tool for the comparison of datasets produced by HTP methods, allowing the quantification of the quality of our experiments in terms of sensitivity, specificity and related metrics (for example true positive rate and false discovery rate (FDR)).

Within the field of cilia and ciliopathy research, existing sets of databases, such as Cildb [2] and Cilia Proteome [3], are already widely consulted and represent an immense asset to ciliary research. This is reflected by the frequency of use of these resources by many cilia research groups (cited 14 and 140 times, respectively, in Thomson Reuters Web of Knowledge, 22 May 2013). However, all studies contributing data to these databases are considered equally informative despite some studies likely suffering from a higher number of false positives than others. Objective estimation of the quality or predictive power of each dataset would be a valuable addition. Calculating the sensitivity and specificity of each dataset will provide an objective indicator of whether to include or exclude datasets for a particular purpose, or how to weigh their contribution in Bayesian data integration. Additionally, comparison of datasets to the SCGS can also facilitate determination of objective cut-off thresholds via receiver operator characteristic (ROC) curves. With the SCGS, we deliver a valuable resource to scientists in the wider field of cilia biology and anticipate a pivotal role for the SCGS in our multi-centre systems biology approach.

### The SYSCILIA gold standard of ciliary genes

**Figure 1:**
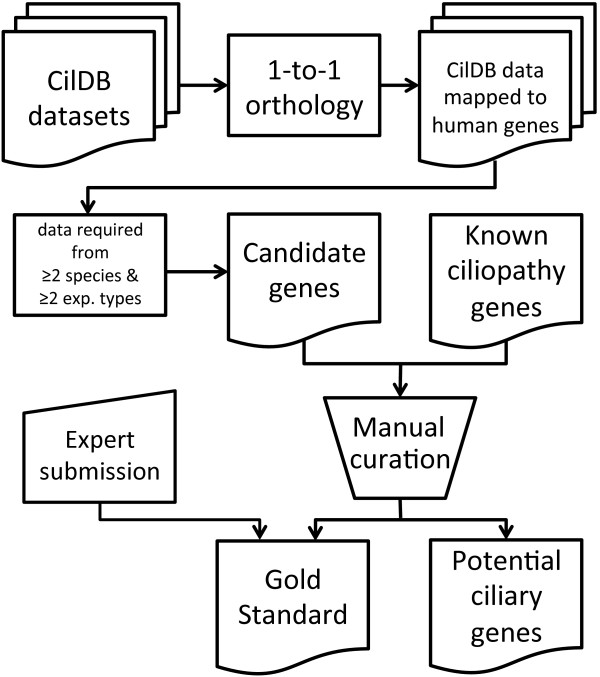
Flow diagram describing the processes to create the SCGS.

As a statistical tool, the SCGS needs to be a high-confidence list of sufficient size, but does not need to be comprehensive; the SCGS does not need to contain all possible ciliary genes to be effective. In order to obtain the most reliable results, the SCGS preferably needs to be free of experimental or other biases and contain no incorrectly assigned genes. For this reason, inclusion of genes based solely on recovery by single HTP experiments or sources with similar potentially high FDRs should be avoided; while genes extensively characterized as ciliary genes in individual ‘gene-specific’ publications, or multiple publications, are highly desirable. Nevertheless, the advantage of HTP results is that they offer a comprehensive starting point to start assembly, without the need to, for example, scan through the whole human genome for cilium genes. An efficient way of combining detailed expert cilia biology knowledge with the comprehensive nature of HTP experiments is to generate an automatically compiled gene list from potentially high quality datasets, curate it manually and combine it with expert knowledge for genes that were missed in the HTP experiments (Figure 1).

To compile the SCGS we collected 27 ciliary studies [2,4-29] from Cildb [2], which holds the largest collection of ciliary datasets (for an overview of the ciliary datasets see Additional file 1). Only datasets based on experimental methods were considered; datasets based on comparative genomics predictions were excluded. The remaining studies covered nine eukaryotic species. All datasets were mapped to human genes by combining two orthology methods, namely OrthoMCL [30] and InParanoid [31]. We only considered one-to-one orthologues between the species of a given dataset and human to avoid cases where after gene duplication, one of the daughter proteins no longer plays a role in the cilium. We defined one-to-one orthologues as defined by InParanoid when both genes are also contained within the same OrthoMCL orthologous group. If InParanoid did not report any human orthologues for a given gene, then the gene reported by OrthoMCL was taken. OrthoMCL performs better in retrieving distant homologues than InParanoid [32], which, with datasets from the distantly related species *Trypanosoma brucei* and *Chlamydomonas reinhardtii*, is particularly invaluable. All other genes in the datasets were excluded, leaving 3,575 genes. The remaining list was then filtered in two ways: data mapped by orthology to a human gene was required to originate from at least two different species and to be shown to be ciliary-related in at least two types of experiments (for example in expression data and proteomics data). A total of 503 genes remained. Finally, a set of 97 medically relevant ciliopathy genes was added from Reeuwijk *et al*. [33]. After removal of overlapping genes, this resulted in a total of 567 potential ciliary genes.

The resulting list of genes was then curated manually. Experts within the SYSCILIA consortium annotated genes as either ‘known-ciliary’, ‘unknown’, or ‘non-ciliary’ based on literature searches. Additionally, members submitted 123 known ciliary genes to this list. Genes were considered ciliary if evidence was published for ciliary localization (including basal body), function in ciliogenesis (including cilium-specific transcription) and involvement in ciliopathies. The final SCGS contains 303 curated ciliary genes.

**Figure 2:**
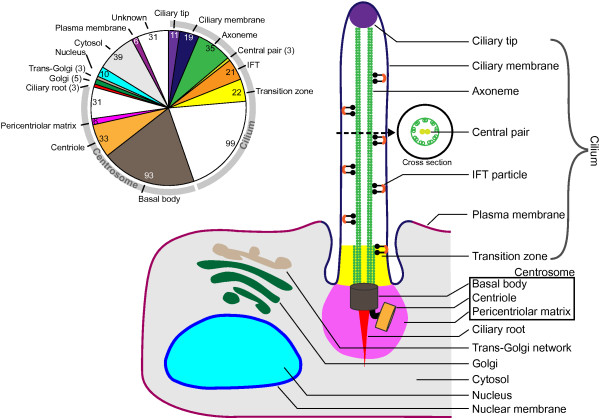
**Schematic overview of ciliary components annotated in the gold standard.** The schematic depiction of the eukaryotic cilium and its components as annotated in the SCGS, based on Basten *et al*. [36]. The pie chart represents the occurrence of ciliary component localization in the SCGS. The numbers in the individual colors represent the number of individual entries for each location. Note that many genes have been ascribed multiple localizations. SCGS, SYSCILIA gold standard.

We are confident that, by combining experimental datasets, a good proportion of the SCGS can be retrieved by commonly used experimental methods. By requiring at least two types of experimental evidence we limit inclusion of experimental biases particular for one type of experiment, like mass spectrometry, which often fails to retrieve membrane proteins [34]. We put effort into annotating the localization of each gene in the SCGS and the SCGS covers all the cilium components (Figure 2). These annotations can be used to quickly compile subsets based on localization.

## Conclusion

Currently, the SCGS is actively used within our consortium for purposes ranging from optimization of experimental methods, to training and evaluating of bioinformatics tools, and as a reference resource. Because of its broad use and importance to the cilia community, we have made the SCGS publicly available (see Additional file 2 and http://www.syscilia.org/goldstandard.shtml). Our list of known ciliary genes is not exhaustive and we expect that the number of newly identified ciliary genes will increase greatly over the next two years. The high stringency applied to the filtering of datasets has led to a small but high-confidence dataset, which we will continue to expand and improve on the basis of novel published cilium genes. Regular updates of the SCGS can be accessed at our consortium website. For many of the metrics discussed above a negative control dataset is also required, that is a list of validated non-ciliary genes. We will also endeavor to make a negative control dataset available in the future. However, it is hard to definitively prove that a gene is never cilia-associated and some genes assigned as negative controls will likely change with new insights. A negative set is therefore volatile; nevertheless SYSCILIA has also recently published such a resource for negative protein-protein interactions [35].

We invite everyone to contribute or curate new and known ciliary genes, to combine and further our collective knowledge on ciliary biology, and use the SCGS to enhance research.

## Availability of supporting data

The SYSCILIA gold standard is provided as an excel file in the supplementary material and online at http://www.syscilia.org/goldstandard.shtml.

## Abbreviations

FDR: False discovery rate; HTP: High-throughput; ROC: Receiver operator characteristic; SCGS: SYSCILIA gold standard; siRNA: Small interfering RNA

## Competing interests

The authors declare that they have no competing interests.

## Authors’ contributions

TJPD and the SYSCILIA Study Group designed the research; TJPD and GW performed the research; TJPD, GW and the SYSCILIA Study Group curated (analysed) the data; and TJPD, GW, GGS, MAH and RHG generated the paper and figures. All authors read and approved the final manuscript.

## Supplementary Material

Additional file 1 Table of ciliary datasets used to compile the gene list, and curate the SCGS and references. Click here for file

Additional file 2 Excel spread sheet of the SYSCILIA gold standard version 1 (SCGSv1), listing 303 curated genes involved in ciliary biology and listing potential ciliary genes. Click here for file
