# Supplementary material for: The SYSCILIA gold standard (SCGSv1) of known ciliary components and its applications within a systems biology consortium
Source: Cilia. 2013 May 31;2:7. doi: 10.1186/2046-2530-2-7 (PMC3674929; doi:10.1186/2046-2530-2-7)
Supplement: Additional file 1 — Table of ciliary datasets used to compile the gene list, and curate the SCGS and references. [file 2046-2530-2-7-S1.docx]

# Additional file 1: Table of ciliary datasets.

Table of ciliary datasets used to compile the gene list used to curate the SCGS and references.

| Study | Experiment types | Species | Reference |
| --- | --- | --- | --- |
| Blacque et al. 2005 | expression, xbox | Caenorhabditis elegans | [1] |
| Chen et al. 2006 | expression | Caenorhabditis elegans | [2] |
| Efimenko et al. 2005 | xbox | Caenorhabditis elegans | [3] |
| Boesger et al. 2009 | proteomics | Chlamydomonas reinhardtii | [4] |
| Keller et al. 2005 | expression, proteomics | Chlamydomonas reinhardtii | [5] |
| Pazour et al. 2005 | expression, Proteomics | Chlamydomonas reinhardtii | [6] |
| Stolc et al. 2005 | expression | Chlamydomonas reinhardtii | [7] |
| Reinders et al. 2006 | proteomics | Dictyostelium discoideum | [8] |
| Laurencon et al. 2007 | xbox | Drosophila melanogaster | [9] |
| Muller et al. 2010 | proteomics | Drosophila melanogaster | [10] |
| Kim et al. 2010 | ciliogenesis_modulation | Homo sapiens | [11] |
| Kubo et al. 2008 | expression | Homo sapiens | [12] |
| Nogales-Cadenas et al. 2006 | curation_centrosome | Homo sapiens | [13] |
| Ostrowski et al. 2002 | proteomics | Homo sapiens | [14] |
| Ross et al. 2007 | expression | Homo sapiens | [15] |
| Cao et al. 2008 | proteomics | Mus musculus | [16] |
| Liu et al. 2007 | proteomics | Mus musculus | [17] |
| McClintock et al. 2008 | expression | Mus musculus | [18] |
| Arnaiz et al. 2009 | proteomics | Paramecium tetraurelia | [19] |
| Arnaiz et al. 2010 | expression | Paramecium tetraurelia | [20] |
| Mayer et al. 2008 | proteomics | Rattus norvegicus | [21] |
| Mayer et al. 2009 | proteomics | Rattus norvegicus | [22] |
| Wigge et al. 1998 | proteomics | Saccharomyces cerevisiae | [23] |
| Kilburn et al. 2007 | proteomics | Tetrahymena thermophila | [24] |
| Smith et al. 2005 | proteomics | Tetrahymena thermophila | [25] |
| Broadhead et al. 2006 | proteomics | Trypanosoma brucei TREU 927 | [26] |
| Stubbs et al. 2008 | expression | Xenopus laevis | [27] |

# References

1. Blacque OE, Perens EA, Boroevich KA, Inglis PN, Li C, Warner A, Khattra J, Holt RA, Ou G, Mah AK, McKay SJ, Huang P, Swoboda P, Jones SJM, Marra MA, Baillie DL, Moerman DG, Shaham S, Leroux MR: **Functional genomics of the cilium, a sensory organelle.** *Current biology : CB* 2005, **15**:935–41.

2. Chen N, Mah A, Blacque OE, Chu J, Phgora K, Bakhoum MW, Newbury CRH, Khattra J, Chan S, Go A, Efimenko E, Johnsen R, Phirke P, Swoboda P, Marra M, Moerman DG, Leroux MR, Baillie DL, Stein LD: **Identification of ciliary and ciliopathy genes in Caenorhabditis elegans through comparative genomics.** *Genome biology* 2006, **7**:R126.

3. Efimenko E, Bubb K, Mak HY, Holzman T, Leroux MR, Ruvkun G, Thomas JH, Swoboda P: **Analysis of xbx genes in C. elegans.** *Development (Cambridge, England)* 2005, **132**:1923–34.

4. Boesger J, Wagner V, Weisheit W, Mittag M: **Analysis of flagellar phosphoproteins from Chlamydomonas reinhardtii.** *Eukaryotic cell* 2009, **8**:922–32.

5. Keller LC, Romijn EP, Zamora I, Yates JR, Marshall WF: **Proteomic analysis of isolated chlamydomonas centrioles reveals orthologs of ciliary-disease genes.** *Current biology : CB* 2005, **15**:1090–8.

6. Pazour GJ, Agrin N, Leszyk J, Witman GB: **Proteomic analysis of a eukaryotic cilium.** *The Journal of cell biology* 2005, **170**:103–13.

7. Stolc V, Samanta MP, Tongprasit W, Marshall WF: **Genome-wide transcriptional analysis of flagellar regeneration in Chlamydomonas reinhardtii identifies orthologs of ciliary disease genes.** *Proceedings of the National Academy of Sciences of the United States of America* 2005, **102**:3703–7.

8. Reinders Y, Schulz I, Gräf R, Sickmann A: **Identification of novel centrosomal proteins in Dictyostelium discoideum by comparative proteomic approaches.** *Journal of proteome research* 2006, **5**:589–98.

9. Laurençon A, Dubruille R, Efimenko E, Grenier G, Bissett R, Cortier E, Rolland V, Swoboda P, Durand B: **Identification of novel regulatory factor X (RFX) target genes by comparative genomics in Drosophila species.** *Genome biology* 2007, **8**:R195.

10. Müller H, Schmidt D, Steinbrink S, Mirgorodskaya E, Lehmann V, Habermann K, Dreher F, Gustavsson N, Kessler T, Lehrach H, Herwig R, Gobom J, Ploubidou A, Boutros M, Lange BMH: **Proteomic and functional analysis of the mitotic Drosophila centrosome.** *The EMBO journal* 2010, **29**:3344–57.

11. Kim J, Lee JE, Heynen-Genel S, Suyama E, Ono K, Lee K, Ideker T, Aza-Blanc P, Gleeson JG: **Functional genomic screen for modulators of ciliogenesis and cilium length.** *Nature* 2010, **464**:1048–51.

12. Kubo A, Yuba-Kubo A, Tsukita S, Tsukita S, Amagai M: **Sentan: a novel specific component of the apical structure of vertebrate motile cilia.** *Molecular biology of the cell* 2008, **19**:5338–46.

13. Nogales-Cadenas R, Abascal F, Díez-Pérez J, Carazo JM, Pascual-Montano A: **CentrosomeDB: a human centrosomal proteins database.** *Nucleic acids research* 2009, **37**:D175–80.

14. Ostrowski LE, Blackburn K, Radde KM, Moyer MB, Schlatzer DM, Moseley A, Boucher RC: **A proteomic analysis of human cilia: identification of novel components.** *Molecular & cellular proteomics : MCP* 2002, **1**:451–65.

15. Ross AJ, Dailey LA, Brighton LE, Devlin RB: **Transcriptional profiling of mucociliary differentiation in human airway epithelial cells.** *American journal of respiratory cell and molecular biology* 2007, **37**:169–85.

16. Cao W, Gerton GL, Moss SB: **Proteomic profiling of accessory structures from the mouse sperm flagellum.** *Molecular & cellular proteomics : MCP* 2006, **5**:801–10.

17. Liu Q, Tan G, Levenkova N, Li T, Pugh EN, Rux JJ, Speicher DW, Pierce EA: **The proteome of the mouse photoreceptor sensory cilium complex.** *Molecular & cellular proteomics : MCP* 2007, **6**:1299–317.

18. McClintock TS, Glasser CE, Bose SC, Bergman DA: **Tissue expression patterns identify mouse cilia genes.** *Physiological genomics* 2008, **32**:198–206.

19. Arnaiz O, Malinowska A, Klotz C, Sperling L, Dadlez M, Koll F, Cohen J: **Cildb: a knowledgebase for centrosomes and cilia.** *Database* 2009, **2009**:bap022.

20. Arnaiz O, Goût J-F, Bétermier M, Bouhouche K, Cohen J, Duret L, Kapusta A, Meyer E, Sperling L: **Gene expression in a paleopolyploid: a transcriptome resource for the ciliate Paramecium tetraurelia.** *BMC genomics* 2010, **11**:547.

21. Mayer U, Ungerer N, Klimmeck D, Warnken U, Schnölzer M, Frings S, Möhrlen F: **Proteomic analysis of a membrane preparation from rat olfactory sensory cilia.** *Chemical senses* 2008, **33**:145–62.

22. Mayer U, Küller A, Daiber PC, Neudorf I, Warnken U, Schnölzer M, Frings S, Möhrlen F: **The proteome of rat olfactory sensory cilia.** *Proteomics* 2009, **9**:322–34.

23. Wigge PA, Jensen ON, Holmes S, Souès S, Mann M, Kilmartin J V: **Analysis of the Saccharomyces spindle pole by matrix-assisted laser desorption/ionization (MALDI) mass spectrometry.** *The Journal of cell biology* 1998, **141**:967–77.

24. Kilburn CL, Pearson CG, Romijn EP, Meehl JB, Giddings TH, Culver BP, Yates JR, Winey M: **New Tetrahymena basal body protein components identify basal body domain structure.** *The Journal of cell biology* 2007, **178**:905–12.

25. Smith JC, Northey JGB, Garg J, Pearlman RE, Siu KWM: **Robust method for proteome analysis by MS/MS using an entire translated genome: demonstration on the ciliome of Tetrahymena thermophila.** *Journal of proteome research* 2005, **4**:909–19.

26. Broadhead R, Dawe HR, Farr H, Griffiths S, Hart SR, Portman N, Shaw MK, Ginger ML, Gaskell SJ, McKean PG, Gull K: **Flagellar motility is required for the viability of the bloodstream trypanosome.** *Nature* 2006, **440**:224–7.

27. Stubbs JL, Oishi I, Izpisúa Belmonte JC, Kintner C: **The forkhead protein Foxj1 specifies node-like cilia in Xenopus and zebrafish embryos.** *Nature genetics* 2008, **40**:1454–60.
